# Breast Cancer and Its Relationship with the Microbiota

**DOI:** 10.3390/ijerph15081747

**Published:** 2018-08-14

**Authors:** Mariana F. Fernández, Iris Reina-Pérez, Juan Manuel Astorga, Andrea Rodríguez-Carrillo, Julio Plaza-Díaz, Luis Fontana

**Affiliations:** 1 Department of Radiology, School of Medicine, and Biomedical Research Center, University of Granada, 18071 Granada, Spain; juanmastorga@correo.ugr.es (J.M.A.); andrearc@ugr.es (A.R.-C.); 2 Health Research Institute of Granada (ibs.GRANADA), 18010 Granada, Spain; jrplaza@ugr.es; 3 Spanish Consortium for Research on Epidemiology and Public Health (CIBERESP), 28029 Madrid, Spain; irisreina@ugr.es; 4 Department of Biochemistry and Molecular Biology II, School of Pharmacy, University of Granada, 18071 Granada, Spain; 5 Institute of Nutrition and Food Technology “José Mataix”, Biomedical Research Center, Parque Tecnológico Ciencias de la Salud, University of Granada, Armilla, 18100 Granada, Spain

**Keywords:** breast cancer, estrobolome, estrogens, microbiota

## Abstract

The microorganisms that live symbiotically in human beings are increasingly recognized as important players in health and disease. The largest collection of these microorganisms is found in the gastrointestinal tract. Microbial composition reflects both genetic and lifestyle variables of the host. This microbiota is in a dynamic balance with the host, exerting local and distant effects. Microbial perturbation (dysbiosis) could contribute to the risk of developing health problems. Various bacterial genes capable of producing estrogen-metabolizing enzymes have been identified. Accordingly, gut microbiota is capable of modulating estrogen serum levels. Conversely, estrogen-like compounds may promote the proliferation of certain species of bacteria. Therefore, a crosstalk between microbiota and both endogenous hormones and estrogen-like compounds might synergize to provide protection from disease but also to increase the risk of developing hormone-related diseases. Recent research suggests that the microbiota of women with breast cancer differs from that of healthy women, indicating that certain bacteria may be associated with cancer development and with different responses to therapy. In this review, we discuss recent knowledge about the microbiome and breast cancer, identifying specific characteristics of the human microbiome that may serve to develop novel approaches for risk assessment, prevention and treatment for this disease.

## 1. Introduction

The incidence of breast cancer (BC) worldwide has risen to unprecedented levels in recent decades, making it the major cancer of women in many parts of the world nowadays [1]. It is not only the most frequently diagnosed cancer (excluding non-melanoma skin cancers) among women worldwide, affecting one in eight women during their lifetime, but also one of the leading causes of cancer mortality in women, with more than 0.5 million deaths in 2012 (6.4% of total cancer deaths, globally, and 15.4% in more developed countries) [2,3]. In 2012, BC accounted for approximately 1.7 million new cases (25% of total cancer incidence, globally). Moreover, between 2008 and 2012 the incidence of BC increased by 20%, and mortality by 14% [4]. Rates are generally high in North America, Australia-New Zealand, and Northern and Western Europe; intermediate in Central and Eastern Europe, Latin America, and the Caribbean; and low in most of Africa and Asia [5].

Cancer in general is a complex disease, where a multitude of genomic and physiological alterations occur incessantly in the tumor tissue, contributing to the complexity of disease treatment and management. For this reason, and regardless of efforts that have been achieved by extensive research, the precise etiology for BC is still unknown, but the combination of genetic, epigenetic, and environmental factors has been identified. Nevertheless, those genetic-epigenetic determinants and well-established risk factors only could explain a limited amount of the global burden of this disease [6]. As a result, the origin of a vast majority of breast cancer cases (estimated to be as high as 70%) still remains unknown [7]. Therefore, it is crucial to understand how these sporadic breast cancers arise in order to develop preventative strategies against this devastating disease.

Apart from genetic factors, such as inherited loss of the *BRCA1*/*2* gene, several environmental and lifestyle components have also been strongly related to BC. Although diet, alcohol, and radiation have all been associated with increased incidence, the main identified risk factors are life exposure to hormones including physiological variations associated with puberty, pregnancy, menopause, personal choice of use of hormonal contraceptives and/or hormone replacement therapy [1]. BC risk is directly related to higher levels of endogenous estrogens and differences in estrogen metabolism, especially among postmenopausal women [8,9]. The decline associated with menopause in age-specific incidence rates, known as the Clemmesen hook, is widely observed among women around the world [10,11]. Reproductive history, alcohol intake, obesity, and use of hormone therapy exert their effects, at least in part, by modifying the time and intensity of the exposure of the mammary gland to steroidal hormones [12]. This hormonal regulation manifests, both clinically and molecularly, as distinct subtypes: Triple negative, HER2 positive (HER2+), and ER positive (ER+) [13].

Bacterial communities within the host could be one additional environmental factor related to BC, which has been only recently considered in sporadic breast cancers of unknown etiology. In the recent years, there has been a strong interest in fully characterizing the microbiota associated with different parts of the body under different health conditions, due to the fact that different published studies have shown that bacterial communities vary across body habitats, establishing complex interactions between bacteria and the host [14,15]. These studies have been possible with the use of deep-sequencing technologies (for example, pyrosequencing technique, which provides a qualitative survey of relative abundances of microbiota, and quantitative polymerase chain reaction (qPCR) which may determine quantitative differences), and also due to the findings from the Human Microbiome Project analyses, which has demonstrated that the diversity and abundance of the signature microbes in each habitat varied among healthy subjects, with strong niche specialization both within and among individuals [14].

The terms microbiota and microbiome are often used as synonyms, but express two different meanings: whereas microbiota refers to the totality of microbes (bacteria, archaea, fungi, viruses and protozoa) in a particular environment, in other words, it refers to taxonomy and abundance of community members, the microbiome is the totality of genomes of a microbiota and it is often used to describe the entity of microbial traits (functions) encoded by a microbiota [16]. The improvement of DNA-RNA sequencing methods has made possible to group microbiota composition into clusters, known as Operational Taxonomic Units (OTUs), based on genetic sequence similarities of specific taxonomic marker genes. Each OTU may belong to different phyla, but new methods that resolve amplicon sequence variants (ASVs) from Illumina-scale amplicon data without imposing the arbitrary dissimilarity thresholds that define molecular OTUs, have been recently developed [17,18,19,20,21,22]. ASV methods infer the biological sequences in the sample prior to the introduction of amplification and sequencing errors and distinguish sequence variants differing by as little as one nucleotide [23].

The human microbiota is the term applied to the universe of microbes that live in different habitats of our body (mostly the gut, skin, vagina and mouth, but also the nose, conjunctiva, pharynx and urethra, among others). The microbiota of each organ is distinct, and there is an important and functionally relevant inter-individual variability of microbiomes, which makes them a potential determinant of disease (including cancer) development [14]. Human bacterial composition contributes and affects different diseases including metabolic disorders, inflammatory and autoimmune diseases, and allergy [24], and even diseases where microbiota involvement seems unlikely [25,26]. The microbiota has been also implicated in cancer development progression and aggressiveness at a variety of body sites [25,27] including stomach [28], colon [29], liver [30], lung [31], and skin [32]. Epidemiologic studies suggest that the human microflora contributes to 16–18% or more of worldwide malignancies [33].

This review discusses important questions such as the role of the human microbiota in BC development, its ability to modulate inflammation, immunity and metabolism, and the possibility that both intestinal and local microbes could affect cancer prevention and be a new target for therapeutic approaches, thus improving the prognosis and quality of life of breast cancer patients.

## 2. Gut Microbiota

The human gastrointestinal tract is colonized by a complex and diverse community composed of trillions of individual microorganisms comprising 1014 heterogeneous species of bacteria, viruses, archaea, and fungi [24]. Their collective bacterial genome harbors approximately 150-fold more genes than the human genome [14]. So far, the human gastrointestinal tract is the best investigated microbiota and is serving as a model for understanding host–microbiota interactions and disease. Main phyla of the gut microbiota are *Firmicutes*, *Bacteriodetes*, *Actinobacteria*, *Proteobacteria*, *Fusobacteria*, *Verrucomicrobia*, *Tenericutes* and *Lentisphaerae*; and main genera are *Bacteroides*, *Clostridium*, *Faecalibacterium*, *Eubacterium*, *Ruminococcus*, *Peptococcus*, *Peptostreptococcus*, *Lactobacillus*, *Streptococcus*, *Streptomyces* and *Bifidobacterium* [25].

The human gut microbiota is helpful in physiologic activities such as digestion, metabolism, and homeostasis, and plays important roles in human health. For example, it is essential in digestion and absorption of indigestible carbohydrates for humans (dietary fiber), production of vitamins B and K, metabolism of endogenous and exogenous compounds, and immunity. It is also actively involved in innate and cell-mediated immunity, helps to maintain intestinal barrier function, and assists with an appropriate immune response against pathogenic microbes [25,34].

The composition of the intestinal microbiota is determined by several internal and external factors, including age, race, diet, maternal colonization, and hygiene, as well as host genetics and environmental exposures to xenobiotics and antibiotics [34,35]. For instance, dietary fiber has been shown to affect the composition of gut bacteria and reduce intestinal β-glucuronidase activity, resulting in a decreased deconjugation and reabsorption of estrogens [36]. Additionally, some individual factors such as stress, travelling, or pharmacological treatment or drugs, can also directly and rapidly produce changes [25]. This complexity represents a challenge to the goal of phenotyping all these populations in each individual and comparing them with others [25].

A symbiotic relationship (referred to as normobiosis) between host and microbiota is critical to maintaining a balance (homeostasis) in the gut. This symbiotic relationship confers benefits to the host in many key aspects of life. However, any perturbation of the normal microbiome content that disrupts this relationship, the so-called “dysbiosis”, may result in detrimental consequences for the host, and promote different diseases [27]. Moreover, it is known that differential abundances of certain microorganisms in the composition of the microbial community, and/or a discrete presence of some bacterial species can exert pathogenic effects that encourage disease development. Thus, *Helicobacter pylori* infection is known to promote gastric cancer and gastric mucosal-associated lymphoid tissue (GALT) lymphoma [37], although *H. pylori*-mediated protection against extra-gastric immune and inflammatory disorders has been described [38]. This pathogen bacterium is classified as a carcinogen by the International Agency for Research on Cancer (IARC).

In contrast to gastric carcinogenesis, recent evidence suggests that human disease is attributable not only to single pathogens but also to global changes in the microbiota [25]. In fact, altered host–gut microbiota interactions, caused by dysbiosis, seem to play an important role in colorectal carcinogenesis [39]. In this context, decreasing overall diversity has been associated with colorectal cancer [40]. Nevertheless, in colon cancer, the overabundance of a single bacterial species (*Fusobacterium nucleatum*) has been correlated with disease and with increased likelihood of lymph node metastasis [41]. But the bacterium *Bacteroidetes fragilis* exerts a protective effect against colitis by modulating inflammatory immune responses in the gut [42].

The gastrointestinal tract exerts both local and long-distance effects in other parts of the body. The liver does not contain a known microbiota and is a good example of a cancer that is promoted by dysbiotic microbiota through long-distance mechanisms. Moreover, intestinal bacteria may promote liver cancer through proinflammatory microorganism-associated molecular patterns (MAMPs) and bacterial metabolites, both of which reach the liver via the portal vein [30,43].

In addition to modulating inflammation and influencing genomic stability of host cells through the deregulation of different signals/pathways, gut microbiota has also been related with cancer progression by affecting metabolic pathways of estrogens through enterohepatic circulation [25]. In this regard, it has been proposed that certain gut microbes may play a role in breast carcinogenesis by promoting antitumor immunity and immune surveillance, and/or by modulating systemic estrogen levels [44,45]. BC associations with estrogen levels could reflect differences among individuals in their intestinal microbial communities [46], as shown 50 years ago by Adlercreutz and collaborators, who demonstrated one of the fundamental roles of the gut microbiota [47]. More recently, Fuhrman and co-workers demonstrated that postmenopausal estrogen metabolism is associated with microbial diversity [13].

## 3. Is There a Link between Gut Microbiota and Breast Cancer?

The link between intestinal microbiota dysbiosis and breast cancer has been investigated in several case-control studies, and many hypotheses have been suggested, some of which underline the possible decrease in the metabolic ability of the microbiota and a weakness of the immune system [48]. The results of clinical studies on the relationship between gut microbiota and breast cancer are summarized in Table 1. Most studies rely on the sequencing of a specific region within the bacterial 16S rRNA gene, with the exception of several studies that are based on either qPCR or a DNA array (PathoChip) (Table 1).

The studies targeting at the relationship between BC and gut microbiota are quite limited so far. In 2011, Plottel and Blaser [49] extensively discussed the so-called “estrobolome”, the collection of the enteric bacterial genes whose products metabolize estrogen and its metabolites. Perturbations in the microbiota/estrobolome can therefore lead to elevated levels of circulating estrogens and its metabolites, thereby increasing the risk of BC. Indeed, clinical studies have identified associations between the gut microbiota, and urinary estrogens and estrogen metabolites [13].

The metabolism of estrogens takes places in the liver, where they are conjugated and excreted into the gastrointestinal lumen within the bile; there they are de-conjugated by bacterial β-glucuronidase, and then they are re-absorbed as free estrogens through enterohepatic circulation, getting to different organs like the breast [25]. These metabolites are produced through estrogen metabolism by several bacteria included in *Clostridia* and *Ruminococcaceae* families [25]. In addition, others estrogen-like metabolites can be also produced by oxidative and reductive reactions in the gut and by an induced synthesis of estrogen-inducible growth factors, which might have a carcinogenic potential. Moreover, bacterial β-glucuronidase could participate in the deconjugation of xenobiotics and/or xenoestrogens, leading to their reuptake through the enterohepatic pathway and thus increasing the time they remain in the organism [50]. Many β-glucuronidase bacteria are found in two dominant subgroups, namely the *Clostridium leptum* cluster and the *Clostridium coccoides* cluster, which belong to the *Firmicutes* phylum. The *Escherichia*/*Shigella* bacterial group, a member of the *Proteobacteria* phylum, also possesses β-glucuronidase enzymes [51].

Other studies have focused on the relationship between gut microbiome and BC risk through estrogen-independent pathways [49,52,53]. An enteromammary pathway by which some bacteria from the gut could reach the mammary gland via an endogenous route has been the most studied pathway [54]. A case-control study comparing the fecal microbiota between BC patients and paired controls found significantly altered microbiota composition and less diverse gut bacteria among the postmenopausal BC patients [52]. The gut microbiome of case patients had higher levels of *Clostridiaceae*, *Faecalibacterium*, and *Ruminococcaceae*; and lower levels of *Dorea* and *Lachnospiraceae*. Moreover, the fecal microbiota was less diverse (α-diversity) in cancer patients. Total estrogens correlated with α-diversity in control patients but not in case patients; and, unexpectedly, cancer patients had higher but not statistically significant levels of systemic estrogens. A possible explanation may involve other known breast cancer risk factors such as adiposity and obesity, since in these circumstances the gut microbiota is less diversified [55].

The gut microbiota has been also related to the development of adiposity and obesity [55], and it is known that overweight and obese women have a higher risk of BC compared to healthy weight women, especially during the postmenopausal period [56]. Within the gut, *Firmicutes* and *Bacteroidetes* are the two main phyla involved in the colonic metabolism of indigestible nutrients, including dietary fibers and polyphenols [57]. Several studies have described a higher ratio of *Firmicutes*/*Bacteroidetes* (F/B) in obese versus lean subjects, although with controversial results [58]. Significant differences in the absolute numbers of total bacteria and of three bacterial groups (*Firmicutes*, *Faecalibacterium prausnitzii*, and *Blautia*) were observed in feces as a function of the body mass index (BMI) of women with early-stage breast cancer, with lower number of bacteria in overweight and obese patients [48]. The lower number of *Firmicutes* bacteria observed in overweight subjects contrasted with other clinical studies showing an increased ratio (F/B) with obesity [59], no effect [60], or a decreased ratio [61] in obese patients. The relation between these main phyla and BMI remains unclear [48].

Goedert and co-workers [53] investigated the role of immunity and inflammation in BC risk, and whether the gut microbiota differed in the composition of immune-recognized microbiota in a case-control study. Cases and controls had significantly different composition of the IgA+ microbiota. When they divided cases into those with IgA+ and IgA− microbiota, cancer cases IgA+ had an altered composition, lower richness and α-diversity of their fecal microbiota, significantly more marked than cases with the IgA-negative microbiota, after adjustment for estrogen levels and other variables [53]. Distributions of estrogen metabolites versus parent estrogen (potentially reflecting differences in estrogen metabolism) were essentially identical in cases and controls. Levels of parent estrogens (estrone and estradiol) and major estrogen metabolites were all non-significantly higher in cases compared with controls [53]. Compared to controls, postmenopausal breast cancer women had significant and different estrogen-independent associations with the IgA+ and IgA− gut microbiota, suggesting that the gut microbiota may influence BC risk by altered metabolism, estrogen recycling, and immune pathways.

Bard and colleagues [62] also evaluated the composition of the gut microbiota among BC patients with different clinical characteristics. The majority of the participants were early-stage invasive ductal carcinoma. Patients with grade III cancer (n = 7) had higher absolute numbers of *Blautia* spp. compared with grade I patients (*p* = 0.048). In addition, absolute numbers of *Bifidobacterium* and *Blautia*, and proportions of *Faecalibacterium Prausnitzii* and *Blautia*, varied according to clinical stages, suggesting that gut microbiota may be related with the development and evolution of BC. In this study, significant differences were also observed for absolute numbers of total bacteria and for some studied bacterial groups (*F. Prausnitzii*, *Firmicutes*, *Blautia* and *Egerthella*) according to BMI [62].

*Firmicutes* and *Bacteroidetes* phyla were also the most numerous bacteria observed in the feces of 31 women with early-stage breast cancer [48]. This study evaluated the relationship between the composition of gut microbiota with the clinic-biological characteristics of BC, describing that the total number of *Bacteroidetes*, *Clostridium coccoides* cluster, *C. leptum* cluster, *F. prausnitzii*, and *Blautia* spp. were significantly higher in clinical stage groups II/III than in clinical stages 0/I, and that *Blautia* spp. were higher in the more severe histoprognostic grade cases. Moreover, authors found a significant decline in the abundance of total bacteria and in three bacterial groups (*Firmicutes*, *Faecalibacterium prausnitzii*, and *Blautia*) as a function of the BMI of participants, with lower number of bacteria in overweight and obese patients [48].

## 4. Mammary Microbiota and Breast Cancer

Some authors have also raised the question of the role of the mammary microbiome in modulating the risk of breast cancer development. The hypothesis is that changes in the composition of breast microbiota may also contribute to disease development and progression through several pathways, but it is still unclear whether the host´s microbial differences are a consequence or a cause of this human disease [52,63,67,71]. It is also still unclear whether there is a specific microbial signature (either for the presence of pathogenic strains or the absence of beneficial ones) responsible of breast carcinogenesis. The results of clinical studies on the relationship between mammary microbiota and BC are also included in Table 1.

The human breast is not sterile but contains a diverse and unique community of bacteria, distinct from that found at other body sites, regardless of the location sampled within the breast, age, country of origin, history of pregnancy, presence/absence of breast malignancy, and method of DNA preparation and sequencing technologies [44,63,71]. To date, several findings seem to demonstrate the potential origin of part of the breast tissue microbiome, by translocation from the gastrointestinal tract, in addition to the skin, via the nipple-areolar orifices, nipple-oral contact via lactation and/or sexual contact [66]. In this regard, it has been proposed that this breast microbiome contributes to maintenance of healthy breast tissue by stimulating, for example, resident immune cells, although the type of bacteria and their metabolic activity, such as the ability to degrade carcinogens, may also contribute [44].

Only few studies have characterized which bacteria are present in breast tumor tissue and normal adjacent tissue of women, finding mixed results in the breast tissue microbiota of patients with and without cancer. These investigations are still in their infancy, and the evidence remains unclear on whether a difference exists between tumor and adjacent histologically-normal tissue from cancer patients. Thus, some studies by Urbaniak et al. have shown that bacterial communities do not differ between tumor tissue and normal adjacent tissue, at both population an individual level [63,67]. Nevertheless, these authors found a higher abundance of *Escherichia coli*, known for its cancer-promoting activity, in women with cancer compared with healthy controls.

Xuan et al. [44] also found that the amount of bacteria was not significantly different in paired normal tissue from BC patients and healthy breast tissue from healthy individuals. The number of OTUs detected did not vary between paired normal and tumor tissue, indicating no significant difference in richness [44]. However, breast tumor tissue had significantly reduced amounts of bacteria: the evenness of the communities was significantly different (*p* = 0.01). Of the 1614 OTUs detected, 11 OTUs were differentially abundant (*p* < 0.05). Of those 11 OTUs, 8 were more abundant in paired normal tissue and 3 were more abundant in tumor tissue. Xuan et al. [44] observed differential abundance with the genera *Methylbacterium* and *Sphingomonas* between paired healthy and/or normal adjacent tissue and tumor tissue, suggesting a potential role of those in cancer development. *Methylobacterium radiotolerans* was relatively enriched and the most prevalent bacterium (found in 100% of samples) in tumor tissue, while *Sphingomonas yanoikuyae* was relatively enriched, the most prevalent (found in 95% of samples) and with absolute levels significantly higher in paired normal tissue. *Sphingomonas yanoikuyae* was not present in the corresponding tumor tissue. The relative abundances of these two bacterial species were inversely correlated in paired normal breast tissue but not in tumor tissue, indicating that dysbiosis may be associated with BC. *M. radiotolerans* was detected in all samples, but its absolute levels did not vary significantly between paired normal and tumor tissue, meaning that its higher relative abundance in tumor tissue reflects a decrease in other bacteria present and not an increase in the absolute level of the organism [44].

Most of the works dealing with the microbiome of breast tissue describes a community characterized by a predominance of the phyla *Proteobacteria* and *Firmicutes* [44,63,67], with the exception of one study that found a predominance of *Bacteroidetes* and very little *Proteobacteria* [66]. Urbaniak et al. [63] studied whether there was a specific microbiome within the mammary tissue, and whether the microbes were different in the breast tissue of 81 women from Canada and Ireland, with and without cancer. A diverse population of bacteria was detected within tissue collected from sites all around the breast. *Proteobacteria* and *Firmicutes* were the most abundant phyla in breast tissue, compared with other taxonomic groups. Authors justified these findings due to a probable host microbial adaptation to the fatty acid environment in the breast tissue. The main OTUs found belonged to 7 different phyla, *Proteobacteria*, *Firmicutes*, *Actinobacteria*, *Bacteroidetes*, *Deinococcus-Thermus*, *Verrucomicrobia*, and *Fusobacteria*, with *Proteobacteria* being the most abundant, followed by *Firmicutes* (specifically from the class *Bacilli*). An additional important finding was the geographical difference between the Canadian and Irish breast tissue microbiomes. Thus, the most abundant taxa in the Canadian samples were *Bacillus* (11.4%), followed by *Acinetobacter* (10.0%); and unclassified *Enterobacteriaceae* (30.8%), *Staphylococcus* (12.7%), *Listeria welshimeri* (12.1%), *Propionibacterium* (10.1%), and *Pseudomonas* (5.3%) in the Irish samples [63]. However, these differences may be erroneous as the tissue samples were processed in different laboratories using different protocols and reagents.

Urbaniak et al. [67] also showed that there are different bacterial profiles in breast tissue of healthy women and those with breast cancer [67]. Similarly, Hieken et al. [66] found that the breast microbiome in women with malignant disease was notably different from that of women with benign disease. The comparison of normal adjacent tissue from women with BC and tissue from healthy women showed significantly higher relative abundances of *Prevotella*, *Lactococcus*, *Streptococcus*, *Corynebacterium,* and *Micrococcus* in healthy patients, and *Bacillus*, *Staphylococcus*, *Enterobacteriaceae*, *Comamondaceae*, and *Bacteroidetes* in BC patients. These bacteria had the ability to cause DNA damage in vitro. Additionally, there was a decrease in some lactic acid bacteria, known for their beneficial health effects, including anti-carcinogenic properties [72].

Wang et al. [45] reported no major shifts in overall diversity or microbiota content when they compared 57 patients with breast invasive carcinoma and 21 healthy paired normal tissues from women undergoing breast surgery for cosmetic purposes. In cancer patients, adjacent histologically normal and non-cancer samples showed no significantly differences in Shannon diversity index or number of observed OTUs. These data are consistent with Urbaniak et al.’s findings [67] but disagree with those of Xuan et al. [44]. Nevertheless, cancer patient breast tissue microbiota clustered significantly differently from non-cancer patients (*p* = 0.03), mainly due to the fact that breast tissue in cancer patients exhibited a significantly decreased relative abundance of *Methylobacterium* (while this was increased at the site of the tumor in the study of Xuan et al. [44]; median 0.10 vs. 0.24, *p* = 0.03). These differences were higher in the microbiota of urine from cancer patients, characterized by increased levels of gram-positive organisms including *Corynebacterium*, *Staphylococcus*, *Actinomyces*, and *Propionibacteriaceae*, and decreased abundance of *Lactobacillus*. If these data are confirmed in large cohort studies, it would serve to identify possible sites of dysbiosis distant from the breast tissue as a non-invasive biomarker of breast cancer [45].

In contrast to the aforementioned findings, Hieken et al. [66] identified significantly distinct microbial communities in the normal breast tissue adjacent to invasive cancer compared to that of normal breast tissue adjacent to benign disease. An overview of taxonomic profiles showed that the overal microbiota of breast tissue between the two disease states appear to be similar, dominated by *Bacteroidetes* and *Firmicutes*, the same most abundant phyla reported by Urbaniak et al. [63,67]. Neither α-diversity nor the Shannon index showed significant differences; however, β-diversity revealed that the microbial community in breast tissue adjacent to invasive cancer was significantly different from that of women with benign disease, mainly in rare and/or less abundant lineages. Malignancy correlated with enrichment in taxa of lower abundance including the genera *Fusobacterium*, *Atopobium*, *Gluconacetobacter*, *Hydrogenophaga* and *Lactobacillus*. Authors attributed the age and menopausal status of patients as potentially confounders of the identified differences. These specific genus-level has been reported in association with other epithelial malignancies, including colon cancer [73].

The microenvironment in and around the tumor contains a variety of cell types, including the microbiota. Pathophysiological changes occurring in the cells and in the microbial composition could also have a significant impact on the tumor growth [27]. Microbial contribution to the breast tumor microenvironment has been less investigated [44,45,69]. Thompson and co-workers [69] characterized the breast microbiota in 668 breast tumor tissues and 72 non-cancerous adjacent tissues, from The Cancer Genome Atlas, and reported possible microbial compositional shifts among the disease subtypes. The most prevalent phyla in the tumor sites were *Proteobacteria* (48.0%), *Actinobacteria* (26.3%) and *Firmicutes* (16.2%), findings consistent with previous results from Hieken et al. [66] and Urbaniak et al. [67]. *Mycobacterium fortuitum* and *Mycobacterium phlei* were two of the prevalent species differentially abundant in tumor samples. *Proteobacteria* also showed increased presence among tumor tissues, and *Actinobacteria* in non-cancerous adjacent tissues tissue samples [69].

In addition to breast and tumor tissue, nipple aspirate fluid (a natural secretion produced by breast epithelial cells) collected from breast cancer patients has been shown to have a significantly different microbiota profile compared to that collected from healthy volunteers [74]. One study investigated the breast ductal microbiota by characterizing the microorganisms present in nipple aspirate fluid from BC women (ductal carcinomas) and healthy control women [65]. Authors found that the nipple aspirate fluid community composition was different (β-diversity) in women with BC. Having a history of breast cancer significantly affected the nipple aspirate fluid microbial composition and explained 13.5% of the variation among the samples. The most abundant bacteria phyla were *Firmicutes* (42.1%), *Proteobacteria* (32.9%), and *Bacteroidetes* (14.5%). In nipple aspirate fluid collected from BC, there was relatively higher incidence of the genus *Alistipes.* By contrast, an unclassified genus from the *Sphingomonadaceae* family was relatively more abundant in nipple aspirate fluid from healthy control women [65].

The bacterial composition and diversity of sentinel lymph nodes of BC patients were also investigated by Yazdi and co-workers in 123 sentinel lymph nodes, 123 normal adjacent breast tissue specimens and 5 normal mastectomy samples. Authors showed an increased presence of *Methyl bacterium radiotolerance* in sentinel lymph nodes [68].

Along with the presence of signatures for bacteria, parasites and fungi, a substantial presence of viruses in the breast tumor tissue and/or tumor microenvironment have also been confirmed, and some authors have suggested that many of these viral signatures could be associated with specific breast cancer subtypes [70]. For example, Banerjee and co-workers found predominant viral, bacterial, fungal and parasitic genomic sequences signatures, significantly present in the analysis of 100 triple negative breast cancer samples, which was underrepresented in the controls normal tissue (17 matched and 20 non-matched controls). Bacterial probes included representatives of a number of families (*Actinomycetaceae*, *Caulobacteriaceae*, *Sphingobacteriaceae*, *Enterobacteriaceae*, *Prevotellaceae*, *Brucellaceae*, *Bacillaceae*, *Peptostreptococcaceae*, *Flavobacteriaceae*), some of which have been associated with cancers [64]. When authors investigated the diversity of the microbiome in the four major types of breast cancer (endocrine receptor (ER) positive, triple positive, HER2+ positive and triple negative breast cancers), two distinct and significantly different patterns were detected, one for the triple negative and triple positive breast cancer types, and another for the ER+ and HER2+ positive breast cancer samples, compared to healthy breast control tissue [70]. Additionally, each type of BC held signatures for unique bacterial genera. Nevertheless, there were a number of bacterial families present in all four breast cancer types: predominantly *Proteobacteria* followed by *Firmicutes* and *Actinomyces*.

## 5. Functional Pathways

More important as a cancer risk and promoting factor than the microbiota composition is its functionality. Several possible mechanisms have been proposed to understand the microbial influence on breast cancer.

### 5.1. Regulation of Chronic Inflammation and Immunity

The microbiota might promote malignancy by inducing chronic inflammation, by altering the balance of host cell proliferation and death, and by triggering uncontrolled innate and adaptive immune responses [27]. Increased BC risk has been associated with the presence of chronic, persistent, and dysregulated inflammation [75,76]. For instance, one postulated inflammation-related mechanism for breast cancer is upregulation of cyclooxygenase 2 (COX2) and its product, prostaglandin E2 (PGE), which results in increased aromatase expression in adipose tissue and increased conversion of androgen precursors to estrogens [77,78]. In this regard, the use of some non-steroidal anti-inflammatory drugs has been associated with a reduced risk of estrogen receptor (ER)-positive BC incidence or recurrence [79,80,81,82,83,84,85].

A potential inflammatory biomarker is mucosal secretory immunoglobulin A (IgA), essential in maintaining the integrity of the mucosal barrier by recognizing and regulating the composition of the gut microbial community, therefore reducing their fitness and mitigating the host’s innate immune response [86]. The link between BC and the mucosal secretory IgA has been established [53]. This host mechanism limits the access of intestinal antigens to the circulation and limits the invasiveness of potentially dangerous bacterial species [87]. Besides, the microbiota itself represents a functional luminal barrier [88] by maintaining epithelial cell turnover, by mucin production and by competing for resources, thereby suppressing the growth of pathogens.

Some particular microbiota may also have a role in maintaining healthy breast tissue through stimulation of host inflammatory responses. For instance, the presence of the specific bacterium *S. yanoikuyae* in breast tissue of healthy women was associated with a dramatic reduction in its abundance in the corresponding tumor tissue. This increase may lead to decreased bacterial-dependent immune cell stimulation, resulting in a permissive environment for breast tumorigenesis [44].

Several researches have exanimated the role of microbiota in the regulation of specific processes of immunity in the context of cancer development. For example, *Lactococcus* spp. may modulate cellular immunity by maintaining the cytotoxic activity of resident Natural Killer (NK) cells [89], and *Lactococcus lactis* has been shown to activate vital cells related with tumor growth (murine splenic NK cells), enhancing cellular immunity [90].

### 5.2. Genomic Stability and DNA Damage

Although DNA damage may not be sufficient in itself to promote cancer development, double-strand breaks are the most detrimental type of DNA damage caused by genotoxins, reactive oxygen species, and ionizing radiation [91]. Urbaniak et al. [67] examined the ability to induce DNA double-stranded breaks of all bacteria isolates cultured from normal adjacent tissue of breast cancer patients. They found that several *Escherichia coli* (a member of the *Enterobacteriaceae* family) isolates and one *Staphylococcus epidermidis* isolate displayed this ability through the production of colibactin, which could cause genomic instability. Neither *Bacillus* isolates nor *Streptococcus thermophilus* induced double-strand breaks, thus protecting against DNA, which may be explained by their production of anti-oxidant metabolites that neutralize peroxide and superoxide radicals [72].

### 5.3. Metabolic Function

Some differentially abundant pathways have been investigated in breast cancer patients, mainly involving hormone metabolism (estrogens and progesterone), cysteine and methionine metabolism, glycosyltransferases, fatty acid biosynthesis and metabolism, and C5-branched dibasic acid metabolism [92,93].

Thus, many of the microbes related with BC share the β-glucuronidase enzymatic activity mentioned above, which hinders the conjugation of estrogens, among other compounds, and leaves them as biologically active hormones. The perturbation of this estrobolome has been shown to influence both local and systemic levels of estrogen and its metabolites [13,94]. Malignant tissue in ER+ BC contains higher levels of estrogen metabolites as compared to normal breast tissue, attributed in part to altered intracrine signaling [95]. β-Glucuronidase prevalence was also found in nipple aspirate fluid of breast cancer survivors [65]. Many β-glucuronidase bacteria are found in two dominant subgroups, namely the *Clostridium leptum* cluster and the *Clostridium coccoides* cluster, which belong to the *Firmicutes* phylum. The *Escherichia*/*Shigella* bacterial group, a member of the *Proteobacteria* phylum, also possesses β-glucuronidase enzymes [51]. Some studies have shown positive correlations between the abundance of *Streptococcus* and the presence of β-glucuronidase and/or β-glucosidase enzymes [96].

β-Glucuronidase could also play a major role in the deconjugation of endocrine disrupting chemicals, such as bisphenol-A, increasing the time that they remain in the organism. Some endocrine compounds could induce alterations in the gut microbiota and the metabolites they produce, which may be associated with increased inflammation [97].

## 6. Conclusions

The association between women’s microbiota and BC has been explored to a certain extent to date. However, it is still unclear whether the host’s microbiota is causative or contributory to this disease. It is worth pointing out that most of the data available at this time suggest the role of specific microbial agents present in the breast and/or gut microenvironment on this disease. The exact mechanisms underlying the clinical observations described in this review remain to be fully understood. The studies published so far utilize a wide range of DNA extraction methods, amplification primers, sequencers, bioinformatics pipelines, and patient populations impairing the comparison of data. Therefore, new computational approaches (ASVs) that scale linearly with study size, simple merging between independently processed data sets, and forward prediction including accurate measurement of diversity and applicability to communities lacking deep coverage in reference databases are needed [23].

Further studies examining whole microbiota composition and function would provide vital information on its role in breast health. To define specific microbial signatures for BC patients may also have implications for future diagnostic development as well as for therapeutic interventions [98].

## Figures and Tables

**Table 1:** Human studies dealing with microbiota and breast cancer.

Study	Sampling Materials and Site	Microbiota Detection and OTU Picking Method *	Sample Size	Main Findings
Fuhrman et al., 2014 [13]	Urine and feces of healthy postmenopausal women	Pyrosequencing V1-V2 16S rRNA amplicons, QIIME: Ribosomal Data Project Bayesian classifier, specific method not disclosed	60 healthy postmenopausal women	The composition and diversity of the gut microbiota were associated with patterns of estrogen metabolism. Relative abundances of the order *Clostridiales* and the genus *Bacteroides* were directly and inversely related with the ratio estrogen metabolites to estrogen parents, respectively. Associations were independent of age, body mass index, and other study design factors.
Xuan et al., 2014 [44]	Breast tumor tissue and paired normal adjacent tissue from the same women	Pyrosequencing gDNA amplified 16S V4 rDNA, QIIME: Greengenes database, specific method not disclosed	20 patients with ER+ BC	*Proteobacteria*, *Firmicutes*, *Actinobacteria*, *Bacteroidetes* and *Verrucomicrobia* accounted for 96.6% of the microbiota composition. The amount of bacteria was not significantly different in normal tissue from breast cancer women compared with healthy individuals. However, *Methylobacterium radiotolerans* was the most significantly enriched and the most prevalent (100% of samples) in tumor tissues, and *Sphingomonas yanoikuyae* (95% of samples) in paired normal tissues. The relative abundances of these two bacterial species were inversely correlated in paired normal tissue but not in tumor tissue, indicating that dysbiosis was associated with cancer.
Urbaniak et al., 2014 [63]	Breast tumor tissue from lumpectomies, mastectomies and breast reductions	Ion Torrent V6 16S rRNA sequencing, UCLUST: Taxonomic assignments for each OTU were made by the Ribosomal Database Project SeqMatch tool. The 16S rRNA sequences obtained in this study have been deposited in the Short Read Archive at NCBI.	81 Canadian and Irish women [43 Canadian (11 benign, 27 BC, 5 healthy) and 38 Irish (33 BC, 5 healthy)]	*Proteobacteria* and *Firmicutes* were the most abundant phyla in the whole study population. The most abundant taxa in Canadian samples were *Bacillus* (11.4%) and *Acinetobacter* (10.0%); and unclassified *Enterobacteriaceae* (30.8%) in Irish samples. Authors only found higher abundance of *Escherichia coli* in cases compared with healthy controls.
Bard et al., 2015 [62]	Feces from women with breast cancer	PCR targeting 16S rRNA gene sequences, not specified	32 women with BC [invasive ductal (81%), stage 0 (46.9%), grade II (62.5%), ER/PgR + (80%), HER2 + (15%)]	Absolute numbers of *Bifidobacterium* and *Blautia,* and proportion of *F Prausnitzii* and *Blautia* were significantly different according to clinical stages. Women with grade III had increased absolute numbers of *Blautia* sp. compared to women with grade I. Significant differences were also found in the absolute numbers of total bacteria and some bacterial groups (*F prausnitzii*, *Firmicutes*, *Blautia* and *Egerthella*), according to BMI.
Goedert et al., 2015 [52]	Urine and feces of cases and control women	Illumina sequencing and taxonomy V3-V4 16S rRNA genes, QIIME: OTUs were assigned to taxa by matching to the Ribosomal Data Project naïve Bayesian classifier, specific method not disclosed	48 BC postmenopausal women [ER+ (n = 42), PR+ (n = 37) and HER2+ (n = 5)] and 48 paired control women	Compared with control women, case patients had an altered fecal microbiota composition (β-diversity) and a lower α-diversity, which was estrogen-independent. Relative abundance of several taxa differed between cases and control: case patients had higher levels of *Clostridiaceae*, *Faecalibacterium*, and *Ruminococcaceae*; and lower levels of *Dorea* and *Lachnospiraceae*. Cases presented higher urinary estrogen levels. Total urinary estrogens significantly correlated with α-diversity in controls but not in cases patients.
Banerjee et al., 2015 [64]	Breast tissue from TNBC cases, and from matched and non-matched controls. Controls samples obtained from the adjacent non-cancerous breast tissue of the same patient, and from healthy individuals (non-matched)	PathoChip array	TNBC (n = 100) and matched controls (n = 17) and non-matched controls (n = 20), from the Abramson Cancer Center Tumor Tissue and Biosample.	TNBC samples presented a specific microbial signature of viruses, bacteria, fungi and parasites which was underrepresented in normal tissue. This signature was significantly associated with the cancer samples. In the viral signatures *Herpesviridae*, *Retroviridae*, *Parapoxviridae*, *Polyomaviridae*, *Papillomaviridae* families were detected. In the bacterial signatures the highest prevalence was *Arcanobacterium*
Chan et al., 2016 [65]	Nipple aspirate fluid (NAF) and aerolar breast skin	16S-V4 rRNA gene amplicon sequencing, Mothur: sequences were aligned to SILVA v119, specific method not disclosed	Women with breast ductal cancer (25 cases), and healthy women (23 controls)	The NAF microbial community composition was different in women with BC. These microbes showed β-glucuronidase activity. The most abundant bacteria in NAF samples were those belonging to *Firmicutes*, *Proteobacteria*, *and Bacteroidetes* phyla. In NAF from BC, there was relatively higher incidence of *Alistipes*, and an unclassified genus from the *Sphingomonadaceae* family in NAF from healthy women. The areolar skin microbota in BC and HC were not significantly distinguishable.
Hieken et al., 2016 [66]	Breast tissue and breast skin from patients undergoing non-mastectomy breast surgery for cancer or benign disease	16S V3-V5 rDNA hypervariable taq sequencing, IM-TORNADO: Taxonomy was assigned against a Greengenes reference database, specific method not disclosed	Patients with benign breast disease (n = 13) and invasive breast cancer (n = 15); all ER/PR+, HER-2+ (29%)	Breast tissue had its own microbiome, different from the overlying breast skin. Moreover, breast microbiome in women with cancer was notably different from the breast microbiome of women with benign disease. The microbiome from breast tissue was differentially abundant of phyla *Firmicutes*, *Actinobacteria*, *Bacteroidetes*, and *Proteobacteria*. BC correlated with enrichment in taxa of lower abundance of *Fusobacterium*, *Atopobium*, *Gluconacetobacter*, *Hydrogenophaga* and *Lactobacillus* genera.
Urbaniak et al., 2016 [67]	Breast tissue from women with breast cancer (lumpectomies or mastectomies) or healthy women (breast reductions or enhancements)	16S V6 rRNA amplicon sequencing, QIIME: OTU were made by extracting the best hits from the SILVA database. The 16S rRNA sequences obtained in this study have been deposited in the Short Read Archive at NCBI.	58 women with benign (n = 13) or BC (n = 45), and 23 controls (n = 23)	Bacterial profiles were statistically different in normal adjacent tissue from BC women compared with control tissue. The comparison showed significantly higher relative abundance of *Prevotella*, *Lactococcus*, *Streptococcus*, *Corynebacterium*, and *Micrococcus* in healthy patients, and *Bacillus*, *Staphylococcus*, *Enterobacteriaceae* (unclassified), *Comamondaceae* (unclassified) and *Bacteroidetes* (unclassified) in BC.
Yazdi et al., 2016 [68]	Sentinel lymph nodes and normal adjacent breast tissue	RT-PCR and pyrosequencing	123 sentinel lymph nodes and 123 normal adjacent breast tissue specimens	There were significant differences between lymph cancer nodes and normal samples according to the presence of *Methylobacterium Radiotolerance*.
Luu et al., 2017 [48]	Feces from women with early-stage breast cancer	Real-time qPCR	31 women with BC [ER/PgR+ (90%), HER2+ (15%)]	In the fecal samples, *Firmicutes* and *Bacteroidetes* phyla were the most abundant bacteria. The total number (richness) of *Bacteroidetes*, *Clostridium coccoides* cluster, *C. leptum* cluster, *F. prausnitzii*, and *Blautia* spp. were significantly higher in clinical stage groups II/III than in clinical stages 0/I. *Blautia* spp. was associated with a more severe histoprognostic grades. Moreover, total bacteria and three groups: *Firmicutes*, *Faecalibacterium prausnitzii* and *Blautia* spp. showed lower number in overweight and obese women.
Wang et al., 2017 [45]	Urine, and right and left breast tissue from each control patient, and tumor and ipsilateral adjacent normal breast tissue for cases	Illumina 16S V3-V4 rRNA amplification, UCLUST: OTUs were assigned using Greengenes, specific method not disclosed	50 BC patients and 20 healthy controls	Neither significant difference in overall diversity (Shannon diversity) nor in microbiota content (number of observed OTUs) was detected in breast tissue from cancer or control women. However, significantly decreased relative richness of *Methylobacterium* was found in women with BC. Differences in the urinary microbiota of women with BC were detected, with increased abundance of *Corynebacterium*, *Staphylococcus*, *Actinomyces*, and *Propionibacteriaceae* gram-positive bacteria, and decreased abundance of genus *Lactobacillus*.
Thompson et al., 2017 [69]	Breast tumor tissues and normal adjacent tissues from The Cancer Genome Atlas	16S-V3-V5 rRNA amplified, metagenomeSeq package, specific method not disclosed	668 tumor tissues (HER2+, ER+ and TNC BC) and 72 normal adjacent tissues	The most abundant phyla in breast tissues were *Proteobacteria*, *Actinobacteria*, and *Firmicutes*. In tumor samples, the most predominant phylum was *Proteobacteria*, and *Actinobacteria* in normal tissue. *Mycobacterium fortuitum* and *Mycobacterium phlei* were two of the prevalent species observed differentially abundant in the tumor samples. *Escherichia coli* was also more prevalent in the breast tissues.
Goedert et al., 2018 [53]	Urine and feces from postmenopausal BC cases and control women	16S V4 rRNA gene amplicon sequencing, DADA2 package and SILVA. The 16S rRNA gene sequence data and case–control status have been deposited and are available in the Sequence Read Archive under BioProject ID PRJNA383849	48 BC postmenopausal women [ER+ (n = 42), PR+ (n = 37) and HER2+ (n = 5)] and 48 paired control women	Cases had reduced richness (number of species) and α-diversity, significantly more marked in the IgA-positive gut microbiota. Cases showed higher levels of *Clostridiaceae*, *Faecalibacterium*, and *Ruminococcaceae*; and lower levels of *Dorea* and *Lachnospiraceae*. Cases versus controls showed significant and different estrogen-independent associations with the IgA+ and IgA− gut microbiota. Total urinary estrogens correlated with α-diversity only in controls.
Banerjee et al., 2018 [70]	BC tissues (cases), breast control tissues from healthy individuals (reduction surgeries)	PathoChips array	BC [ER+ (n = 50), HER2+ (n = 34), triple positive (n = 24), TNBC (n = 40)], and normal breast tissue (n = 20)	Unique viral, bacterial, fungal and parasitic signatures were found for each of the BC types. The triple negative and positive samples showed distinct microbial signature patterns than the ER and HER2 positive breast cancer samples. The most prevalent bacterial signatures were for *Proteobacteria* followed by *Firmicutes*. The *Mobiluncus* family was detected in all four types.

BC: breast cancer; DADA2: Divisive Amplicon Denoising Algorithm; EM: estrogens metabolites; ER+: estrogen receptor-positive; HC: healthy control; HER2+: human epidermal growth factor receptor 2-positive; NAF: nipple aspirate fluid; OTUs: operational taxonomic units; PR+: progesterone receptor-positive; QIIME: Quantitative Insights Into Microbial Ecology; TNB: triple negative breast cancer. * All studies with OTU picking method have rarefaction curves assays.
